# Supplementary material for: Comparison of the Cox Proportional Hazards Model and Random Survival Forest Algorithm for Predicting Patient‐Specific Survival Probabilities in Clinical Trial Data
Source: Biom J. 2026 Sep 25;68(5):e70171. doi: 10.1002/bimj.70171 (PMC13615326; doi:10.1002/bimj.70171)
Supplement: Supplementary file 2 [file BIMJ-68-e70171-s002.zip › 2_local_analysis/Byar/figures/cens_03/legend_calibration_curves.pdf]

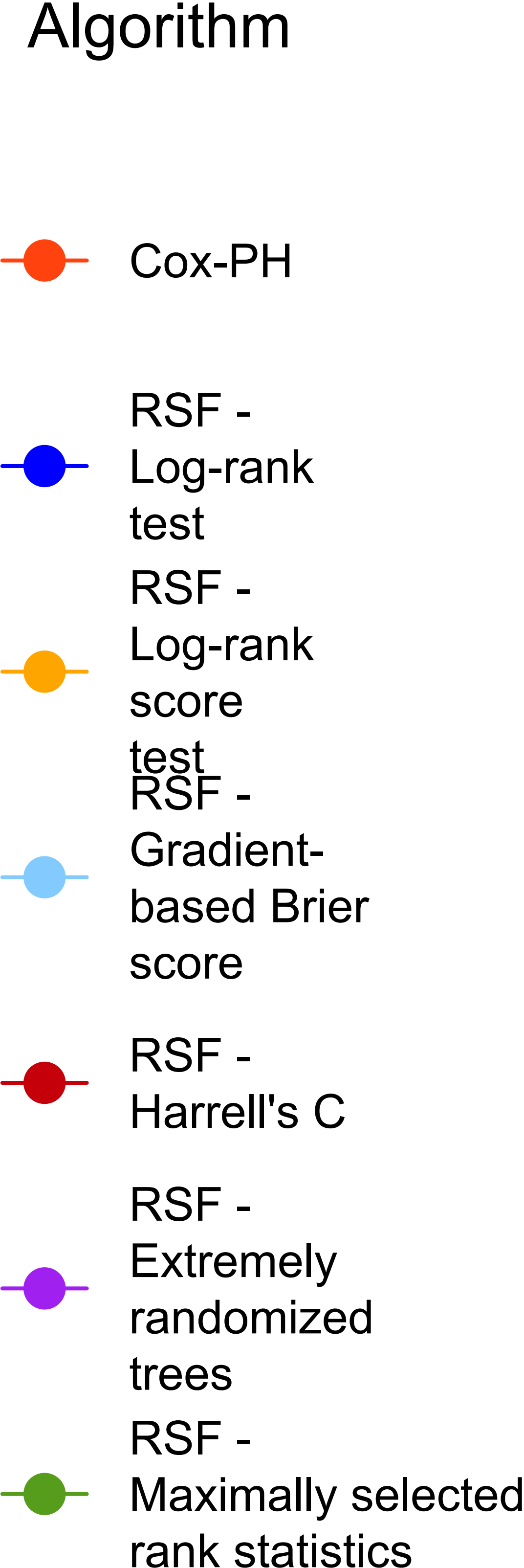
Algorithm
Cox-PH
RSF - Log-rank test
RSF - Log-rank score test
RSF - Gradient-based Brier score
RSF - Harrell's C
RSF - Extremely randomized trees
RSF - Maximally selected rank statistics
